# Overtriage and Undertriage of Children Presenting to the Emergency Department for Behavioral Health

**DOI:** 10.1001/jamanetworkopen.2026.3042

**Published:** 2026-03-24

**Authors:** Jennifer A. Hoffmann, Ashley A. Foster, Christina R. Rojas, Seth Otto, Cody S. Olsen, Huong D. Meeks, Kimberly Denicolo, Aron Janssen, Jacqueline Grupp-Phelan, Elizabeth R. Alpern

**Affiliations:** 1 Division of Emergency Medicine, Ann & Robert H. Lurie Children’s Hospital of Chicago, Chicago, Illinois; 2 Department of Emergency Medicine, University of California, San Francisco; 3 Utah Data Coordinating Center, University of Utah, Salt Lake City; 4 Department of Pediatrics, University of Utah School of Medicine, Salt Lake City; 5 Department of Nursing, Ann & Robert H. Lurie Children’s Hospital of Chicago, Chicago, Illinois; 6 Pritzker Department of Psychiatry and Behavioral Health, Ann & Robert H. Lurie Children’s Hospital of Chicago, Chicago, Illinois

## Abstract

**Importance:**

The Emergency Severity Index (ESI) is the most widely used triage system in US emergency departments (EDs), but its performance in triaging children presenting with behavioral health symptoms is not well studied.

**Objective:**

To assess the frequency of overtriage and undertriage and to identify characteristics associated with both among children presenting to the ED with behavioral health symptoms.

**Design, Setting, and Participants:**

This retrospective cross-sectional study was performed in 15 US EDs participating in the Pediatric Emergency Care Applied Research Network (PECARN) Registry. Participants included children and adolescents aged 5 to 17 years presenting to EDs within the PECARN Registry with a behavioral health chief concern from January 1, 2021, to December 31, 2023. Data were analyzed from July 1, 2024, to January 15, 2026.

**Exposures:**

Sociodemographic and clinical characteristics.

**Main Outcomes and Measures:**

Appropriate triage, overtriage, and undertriage were defined using combinations of first-obtained vital signs, Glasgow Coma Scale, pain score, receipt of emergency medications, distinct resource types used (eg, laboratory tests, imaging studies), and disposition. Multivariable logistic regression assessed characteristics associated with overtriage and undertriage, compared with appropriate triage, adjusting for year and site effects.

**Results:**

A total of 78 411 ED visits by children and adolescents with a behavioral health chief concern (37 328 [47.6%] aged 10-14 years; median age, 14.4 [IQR, 12.4-16.1] years; 47 496 [60.6%] female) were included in the analysis. Of 74 564 visits with nonmissing data, 25 668 (34.4%) were appropriately triaged, 42 589 (57.1%) were overtriaged, and 6307 (8.5%) were undertriaged. The adjusted odds of overtriage were higher for visits by children aged 5 to 9 years (adjusted odds ratio [AOR], 4.43; 95% CI, 4.13-4.76) compared with those aged 10 to 14 years and for visits by non-Hispanic Black (AOR, 1.17; 95% CI, 1.12-1.22) compared with non-Hispanic White patients. The adjusted odds of undertriage were higher for visits by Hispanic patients (AOR, 1.46; 95% CI, 1.31-1.63) and non-Hispanic Black patients (AOR, 1.28; 95% CI, 1.19-1.37) compared with non-Hispanic White patients and for those with Spanish language preference (AOR, 1.31; 95% CI, 1.11-1.54) compared with those preferring English.

**Conclusions and Relevance:**

In this cross-sectional study of children presenting to the ED with behavioral health symptoms, overtriage was common, and the likelihood of overtriage and undertriage differed by sociodemographic characteristics. Prospective studies are needed to assess behavioral health triage practices and to design triage systems that allocate resources accurately and equitably.

## Key Points

**Question:**

What visit characteristics are associated with overtriage and undertriage of children presenting to the emergency department (ED) for behavioral health?

**Findings:**

In this cross-sectional study of 74 564 pediatric visits for behavioral health symptoms across 15 EDs with complete data, 57% were overtriaged and 8% were undertriaged. Overtriage was more likely among visits by younger children, and undertriage was more likely among visits by Hispanic and non-Hispanic Black children than non-Hispanic White children and among visits by children who preferred Spanish compared with English.

**Meaning:**

These findings suggest that overtriage and undertriage of pediatric behavioral health visits in the ED varied by sociodemographic factors, highlighting opportunities to improve triage equity in this population.

## Teaser

This cross-sectional study uses data from the Pediatric Emergency Care Applied Research Network Registry to assess the association of sociodemographic and clinical characteristics with frequency of overtriage and undertriage among children presenting to the emergency department (ED) with mental and behavioral health symptoms.

## Introduction

In emergency medicine, triage differentiates patients who require immediate attention from those who can safely wait for care.^1^ The Emergency Severity Index (ESI), a 5-level acuity scale, is the most used triage system in the US.^2^ Nevertheless, rates of mistriage (ie, resource use discordant with ESI categorization) are high among pediatric ED visits for acute injuries and medical illnesses, which can result in malalignment of resources with patient care needs.^3,4^

Approximately 13% of ED visits by children in the US are for mental and behavioral health (MBH) symptoms,^5^ yet the performance of ESI among these visits is not well studied. The ESI Handbook recommends triaging children as high risk (level 2) if they present a danger to themselves, others, or the environment.^6^ In practice, therefore, many children presenting with MBH symptoms are triaged as level 2,^7^ which may not accurately reflect heterogeneity of MBH presentations. When many children presenting with MBH symptoms are assigned the same triage score, ED care teams may rely on subjective clinical judgment to decide how to allocate limited resources, an approach prone to the influence of implicit bias.^8^ Indeed, disparities in pediatric ED triage by race, ethnicity, and language preference are well described,^9,10,11^ but it is unknown to what extent disparities manifest during triage of children with MBH symptoms specifically. To address these knowledge gaps, we used data from 15 EDs participating in the Pediatric Emergency Care Applied Research Network (PECARN) Registry to assess the frequency of overtriage and undertriage and to identify sociodemographic and clinical characteristics associated with both among children presenting to the ED with MBH symptoms.

## Methods

### Study Design and Data Sources

We conducted a retrospective cross-sectional study using data from 15 US EDs participating in the PECARN Registry from January 1, 2021, to December 31, 2023.^12^ The PECARN Registry consists of electronic health record data from every pediatric ED encounter at participating institutions, harmonized into a deidentified, central repository.^12^ Variables include demographic characteristics, laboratory and radiology results, *International Classification of Diseases, 10th Revision, Clinical Modification* diagnoses, and disposition. Of 17 US EDs participating in the PECARN Registry during the study period, we selected 15 EDs because they had defined lists of chief concerns. Sites included 9 pediatric EDs in quaternary care hospitals and 6 affiliated pediatric EDs located in the following US Census regions: 2 in the Northeast, 3 in the South, 7 in the Midwest, and 3 in the West. This study followed the Strengthening the Reporting of Observational Studies in Epidemiology (STROBE) reporting guideline. The study is a subanalysis of the PECARN Registry, which is approved by the institutional review boards of all study sites and the University of Utah Emergency Medical Services for Children Data Center, which waived the need for informed consent.

### Encounter Inclusion Criteria

We included ED visits by children and adolescents aged 5 to 17 years presenting with an MBH chief concern, with ED length of stay longer than 0 minutes and nonmissing ESI score and ED disposition. We excluded encounters by patients who left without being seen or left against medical advice.

Chief concerns were categorized as MBH if they included terms associated with diagnoses in the *Diagnostic and Statistical Manual of Mental Disorders* (Fifth Edition).^13^ Chief concerns of ingestion or overdose, without intent specified, were categorized as MBH if the patient was 10 years or older, based on the epidemiology of intentional ingestions.^14^ All chief concerns from participating EDs were categorized as MBH vs non-MBH by 2 independent coders (J.A.H. and A.A.F.), with discrepancies resolved through discussion with a third coder (C.R.R.).

### Study Measures

Sociodemographic characteristics, as recorded in the electronic health record, included age group, sex, composite race and ethnicity, language preference (English, Spanish, or other), and primary payer (private, public, or self-pay or other). Race and ethnicity were categorized by ethnicity (Hispanic or non-Hispanic) and then by race within the non-Hispanic group. Due to limited sample sizes, non-Hispanic racial categories were analyzed as Black, White, and other (including American Indian or Alaska Native, Asian, Native Hawaiian or Other Pacific Islander, multiracial, and other race or ethnicity). Race and ethnicity were included in analyses due to previously described racial and ethnic differences in mistriage rates in the pediatric ED.^8,9,11^ Clinical characteristics included arrival mode (by emergency medical services or other); arrival time (daytime [7:00-14:59], evening [15:00-22:59], or overnight [23:00-6:59]); arrival day of week (weekend or weekday); season of visit (school months [September to May] or non–school months [June to August])^15^; arrival volume quintile, reflecting the number of arrivals to the site during the same hour compared with the site-specific hourly median number of visits; assigned ESI triage acuity score (1-5, with 1 representing the highest acuity); time to first clinician evaluation; presence of a complex chronic condition, defined by the patient having any billing diagnosis codes aligned with previously validated criteria during this or a prior ED visit to the same hospital^16^; primary mental health diagnosis group, defined using the Child and Adolescent Mental Health Disorders Classification System, with categories collapsed such that no category included less than 1.5% of the study sample^17^; aggressive behavior as a reason for visit, defined using previously validated diagnosis codes^18^; having a prior MBH visit within 1 year; length of stay in minutes; disposition (admission, observation, or death; transferred; discharged; or other); return ED visits within 7 and 30 days for any reason; and return MBH ED visits within 7 and 30 days.

### Outcome Measures: Overtriage and Undertriage

Aligned with the ESI Handbook Fifth Edition criteria,^6^ we defined appropriate triage, overtriage, and undertriage using combinations of first-obtained vital signs, Glasgow Coma Scale (GCS), and pain score; receipt of emergency medications; number of distinct resource types used (eg, laboratory tests, imaging studies); and disposition (Table 1). Vital signs were defined as high risk if any of the first vital signs (heart rate, respiratory rate, or oxygen saturation) recorded within 2 hours of ED arrival met age-specific criteria (eTable 1 in Supplement 1).^6^ Vital signs were considered stable if both heart rate and respiratory rate did not qualify as high risk and oxygen saturation was 93% or greater or not recorded. GCS was defined by the first score recorded within 2 hours of ED arrival; otherwise, GCS was considered missing. The pain score, standardized from a range of 0 to 10, was defined by the first score recorded within 2 hours of ED arrival; otherwise, the pain score was considered missing. Emergency medications were defined by specific medications delivered via the intravenous, intraosseous, or intramuscular route for pediatric critical care^4^ or pharmacologic restraint^19^ (eTable 2 in Supplement 1).

**Table 1.** Definitions of Undertriage, Appropriate Triage, and Overtriage

Assigned ESI category (number)	Undertriage	Appropriate triage	Overtriage
Lifesaving interventions (1)	NA	Any of the following findings: (1) first Spo_2_ recorded within 2 h of ED arrival <90%; (2) first GSC recorded within 2 h of ED arrival ≤8; (3) required emergency medications within 1 h of ED arrival; or (4) ED disposition of death	No findings indicating appropriate triage and (1) first Spo_2_ recorded within 2 h of ED arrival ≥90% (or not recorded); (2) first GCS recorded within 2 h of ED arrival >9 (or not recorded); and (3) no emergency medications recorded within 1 h of ED arrival
High-risk situation, confused, or severe pain (2)	Any of the following findings: (1) first Spo_2_ recorded within 2 h of ED arrival <90%; (2) first GCS recorded within 2 h of ED arrival ≤8; or (3) required emergency medications within 1 h of ED arrival	No findings indicating undertriage, and any of the following findings: (1) first vital signs recorded within 2 h of ED arrival were high risk (defined as ≥1 of heart rate, respiratory rate, or Spo_2_ qualifying as high risk based on age cutoffs); (2) first pain score recorded within 2 h of ED arrival is ≥7; (3) first GCS recorded within 2 h of ED arrival is 9-15; or (4) required emergency medications >1 h after ED arrival	No findings indicating undertriage or appropriate triage and (1) first vital signs recorded within 2 h of ED arrival were stable (defined as both heart rate and respiratory rate not qualifying as high risk based on age cutoffs, and Spo_2_ ≥93% or not recorded); (2) first pain score recorded within 2 h of ED arrival <7 (or not recorded); (3) first GCS recorded within 2 h of ED arrival is 15 (or not recorded); and (4) no emergency medications recorded during the visit
Not high risk, uses 2 or more resources (3)	Any of the following findings: (1) first vital signs recorded within 2 h of ED arrival were high risk (defined as ≥1 of heart rate, respiratory rate, or Spo_2_ qualifying as high risk based on age cutoffs); (2) first pain score recorded within 2 h of ED arrival is ≥7; (3) first GCS recorded within 2 h of ED arrival <15; (4) Required emergency medications at any time during the visit; or (5) ED disposition of death	No findings indicating undertriage and used ≥2 resources during the visit	No findings indicating undertriage or appropriate triage and (1) first vital signs recorded within 2 h of ED arrival were stable (defined as both heart rate and respiratory rate not qualifying as high risk based on age cutoffs, and Spo_2_ ≥93% or not recorded); (2) first pain score recorded within 2 h of ED arrival is <7 (or not recorded); (3) first GCS recorded within 2 h of ED arrival is 15 (or not recorded); (4) no emergency medications recorded during the visit and (5) used <2 resources
Not high risk, uses 1 resource (4)	Any of the following findings: (1) first vital signs recorded within 2 h of ED arrival were high risk (defined as ≥1 of heart rate, respiratory rate, or Spo_2_ qualifying as high risk based on age cutoffs); (2) first pain score recorded within 2 h of ED arrival is ≥7; (3) first GCS recorded within 2 h of ED arrival <15; (4) required emergency medications at any time during the visit; (5) ED disposition of admission, observation, transfer, or death; or (6) used ≥2 resources during the visit	No findings indicating undertriage and used 1 resource during the visit	No findings indicating undertriage or appropriate triage and (1) first vital signs recorded within 2 h of ED arrival were stable (defined as both heart rate and respiratory rate not qualifying as high risk based on age cutoffs, and Spo_2_ ≥93% or not recorded); (2) first pain score recorded within 2 h of ED arrival is <7 (or not recorded); (3) first GCS recorded within 2 h of ED arrival is 15 (or not recorded); (4) no emergency medications required at any time during the visit; and (5) used no resources during the visit
Not high risk, uses no resources (5)	Any of the following findings: (1) first vital signs recorded within 1 h of ED arrival were high risk (defined as ≥1 of heart rate, respiratory rate, or Spo_2_ qualifying as high risk based on age cutoffs); (2) first pain score recorded within 1 h of ED arrival is ≥7; (3) first GCS recorded within 1 h of ED arrival <15; (4) required emergency medications at any time during the visit; (5) ED disposition of admission, observation, transfer, or death; or (5) used any resources during the visit	No findings indicating undertriage and used no resources during the visit	NA

Abbreviations: ED, emergency department; ESI, Emergency Severity Index; GCS, Glasgow Coma Scale; NA, not applicable; Spo_2_, oxygen saturation by pulse oximeter.

Use of distinct resource types, counted across the entire ED stay, was aligned with ESI Handbook definitions.^6^ Of note, electrocardiography is considered a resource type in the ESI Handbook but was unavailable in the PECARN Registry. Simple procedures and specialty consultation were identified from visit narratives using natural language processing (NLP). Regular expression-based text matching NLP algorithms identified visit narratives with keywords and phrases indicating whether a specialty consultation or 1 of 8 prespecified procedures was performed (eTable 3 in Supplement 1). Clinicians reviewed random samples of narratives in batches and iteratively validated NLP-based algorithms.

### Statistical Analysis

Data were analyzed from July 1, 2024, to January 15, 2026. We described sociodemographic and clinical characteristics and distinct resource types used among ED visits for MBH symptoms, overall and by ESI score categories. ESI scores were grouped for analysis (1 and 2, 3, or 4 and 5) such that no category represented less than 2% of the study sample. We used χ^2^ tests to compare categorical variables and Kruskal-Wallis tests to compare continuous variables by ESI score groupings. We described sociodemographic and clinical characteristics of ED visits classified as overtriaged, undertriaged, and appropriately triaged.

We created 2 site-level scatterplots to assess the association between site demographic composition and rates of appropriate triage: (1) the percentage of visits at each site by patients from racial and ethnic minority groups and the percentage of visits by these patients that were appropriately triaged and (2) the percentage of visits at each site with a language preference other than English and the percentage of these visits that were appropriately triaged. We used multivariable logistic regression to assess sociodemographic and clinical characteristics associated with overtriage and undertriage, each compared with appropriate triage. Informed by prior literature on factors influencing pediatric triage,^3,4,10,20^ the following key sociodemographic and clinical covariates were included in models: age, sex, race and ethnicity, preferred language, arrival time, arrival day of the week, season of visit, arrival volume quintile, presence of a complex chronic condition, prior MBH visit within a year, and ED arrival mode. Models were additionally adjusted for year and site effects. For each variable, the largest category was considered the reference. We calculated adjusted odds ratios (AORs) and 95% CIs and used a 2-sided significance level of .05 to determine statistical significance. We performed all analyses using SAS/STAT software, version 9.4 (SAS Institute Inc).

## Results

### Characteristics of ED Visits by Children for MBH Symptoms

We included 78 411 ED visits by children for MBH symptoms (eFigure 1 in Supplement 1). The most frequently occurring MBH chief concerns are presented in eTable 4 in Supplement 1. Among included visits, 37 328 (47.6%) were by patients aged 10 to 14 years (median age, 14.4 [IQR, 12.4-16.1] years); 47 496 (60.6%), by female patients; 30 909 (39.4%), by male patients; 6 (0.01%), by patients of unknown sex; and 45 926 (58.6%), by publicly insured patients (Table 2). A total of 11 023 visits (14.1%) were by Hispanic patients; 23 379 (29.8%), by non-Hispanic Black patients; 37 088 (47.3%), by non-Hispanic White patients; 6003 (7.7%), by non-Hispanic patients of other race; and 918 (1.2%), by patients of unknown race and ethnicity. The ESI score distribution among ED visits was 220 (0.3%) for level 1, 65 475 (83.5%) for level 2, 6929 (8.8%) for level 3, 4842 (6.2%) for level 4, and 945 (1.2%) for level 5. The most frequently occurring primary mental health diagnosis groups were depressive disorders in 19 975 visits (25.5%) and suicide or self-injury in 17 869 visits (22.8%). The median time to evaluation by a clinician was 35.7 (IQR, 18.0-69.0) minutes (Table 3). The most frequently used resource types were specialty consultation in 32 940 visits (42.0%) and laboratory testing in 19 551 visits (24.9%) (eTable 5 in Supplement 1).

**Table 2.** Demographic and Clinical Presentation Characteristics of Patients With Emergency Department Visits for an MBH Chief Concern

Characteristic	ESI score, No. (%)^a^			
	1 or 2 (n = 65 695)	3 (n = 6929)	4 or 5 (n = 5787)	Overall (N = 78 411)
Age, y				
5-9	6318 (9.6)	1307 (18.9)	962 (16.6)	8587 (11.0)
10-14	31 821 (48.4)	2864 (41.3)	2643 (45.7)	37 328 (47.6)
15-17	27 556 (41.9)	2758 (39.8)	2182 (37.7)	32 496 (41.4)
Sex				
Male	25 380 (38.6)	2905 (41.9)	2624 (45.3)	30 909 (39.4)
Female	40 311 (61.4)	4024 (58.1)	3161 (54.6)	47 496 (60.6)
Unknown	4 (0.01)	0	2 (0.03)	6 (0.01)
Race and ethnicity				
Hispanic	9403 (14.3)	1142 (16.5)	478 (8.3)	11 023 (14.1)
Non-Hispanic Black	19 681 (30.0)	1966 (28.4)	1732 (29.9)	23 379 (29.8)
Non-Hispanic White	30 821 (46.9)	3183 (45.9)	3084 (53.3)	37 088 (47.3)
Non-Hispanic other^b^	5021 (7.6)	569 (8.2)	413 (7.1)	6003 (7.7)
Unknown	769 (1.2)	69 (1.0)	80 (1.4)	918 (1.2)
Preferred language				
English	62 466 (95.1)	6416 (92.6)	5537 (95.7)	74 419 (94.9)
Spanish	2455 (3.7)	413 (6.0)	175 (3.0)	3043 (3.9)
Other	653 (1.0)	88 (1.3)	72 (1.2)	813 (1.0)
Unknown	121 (0.2)	12 (0.2)	3 (0.1)	136 (0.2)
Primary payer				
Public	38 124 (58.0)	4004 (57.8)	3798 (65.6)	45 926 (58.6)
Private	26 727 (40.7)	2811 (40.6)	1799 (31.1)	31 337 (40.0)
Self-pay or other	830 (1.3)	112 (1.6)	183 (3.2)	1125 (1.4)
Unknown	14 (0.02)	2 (0.03)	7 (0.1)	23 (0.03)
Arrival mode				
EMS (ground or air)	18 449 (28.1)	1294 (18.7)	1309 (22.6)	21 052 (26.8)
Other	45 539 (69.3)	5308 (76.6)	4454 (77.0)	55 301 (70.5)
Unknown	1707 (2.6)	327 (4.7)	24 (0.4)	2058 (2.6)
Arrival time				
Daytime (7:00-14:59)	22 673 (34.5)	2908 (42.0)	2028 (35.0)	27 609 (35.2)
Evening (15:00-22:59)	34 397 (52.4)	3298 (47.6)	2958 (51.1)	40 653 (51.8)
Overnight (23:00-06:59)	8625 (13.1)	723 (10.4)	801 (13.8)	10 149 (12.9)
Arrival day of week				
Weekday (Monday to Friday)	53 234 (81.0)	5557 (80.2)	4578 (79.1)	63 369 (80.8)
Weekend (Saturday or Sunday)	12 461 (19.0)	1372 (19.8)	1209 (20.9)	15 042 (19.2)
Season of visit				
Non–school month (June to August)	12 183 (18.5)	1464 (21.1)	1201 (20.8)	14 848 (18.9)
School month (September to May)	53 512 (81.5)	5465 (78.9)	4586 (79.2)	63 563 (81.1)
Arrival volume quintile^c^				
Very low	2071 (3.2)	200 (2.9)	199 (3.4)	2470 (3.2)
Low	5267 (8.0)	573 (8.3)	472 (8.2)	6312 (8.0)
Moderate	11 439 (17.4)	1246 (18.0)	956 (16.5)	13 641 (17.4)
High	19 530 (29.7)	2074 (29.9)	1722 (29.8)	23 326 (29.7)
Very high	27 388 (41.7)	2836 (40.9)	2438 (42.1)	32 662 (41.7)
Time to clinician evaluation, median (IQR), min	35.0 (18.0-66.0)	42.0 (19.8-95.0)	40.0 (20.1-79.7)	35.7 (18.0-69.0)

Abbreviations: ED, emergency department; EMS, emergency medical services; ESI, Emergency Severity Index; MBH, mental or behavioral health.

^a^ Lower ESI scores indicate higher acuity; *P* < .01 for all comparisons except for arrival volume quintile (*P* = .35).

^b^ Includes American Indian or Alaska Native (108 ESI 1 or 2, 10 ESI 3, and 4 ESI 4 or 5), Asian (1099 ESI 1 or 2, 152 ESI 3, and 65 ESI 4 or 5), Native Hawaiian or Other Pacific Islander (83 ESI 1 or 2, 6 ESI 3, and 9 ESI 4 or 5), multiracial (2567, ESI 1 or 2, 229 ESI 3, and 306 ESI 4 or 5), and other race or ethnicity (1175 ESI 1 or 2, 172 ESI 3, and 29 ESI 4 or 5).

^c^ Indicates the number of arrivals to the site during the same hour compared with the site-specific hourly median number of visits.

**Table 3.** Clinical Characteristics, Treatment, and Outcomes of ED Visits by Children and Adolescents Aged 5 to 17 Years for an MBH Chief Concern

Variable	ESI score, No. (%)^a^			
	1 or 2 (n = 65 695)	3 (n = 6929)	4 or 5 (n = 5787)	Overall (N = 78 411)
Complex chronic condition	6408 (9.8)	995 (14.4)	429 (7.4)	7832 (10.0)
Mental health diagnosis group^b^				
Attention-deficit/hyperactivity disorder	1707 (2.6)	111 (1.6)	190 (3.3)	2008 (2.6)
Anxiety disorders	3307 (5.0)	899 (13.0)	661 (11.4)	4867 (6.2)
Autism spectrum disorder	984 (1.5)	64 (0.9)	44 (0.8)	1092 (1.4)
Depressive disorders	17 962 (27.3)	643 (9.3)	1370 (23.7)	19 975 (25.5)
Disruptive, impulse control and conduct disorders	3819 (5.8)	290 (4.2)	719 (12.4)	4828 (6.2)
Substance-related and addictive disorders	1408 (2.1)	379 (5.5)	93 (1.6)	1880 (2.4)
Suicide or self-injury	16 859 (25.7)	342 (4.9)	668 (11.5)	17 869 (22.8)
Trauma and stressor-related disorders	3934 (6.0)	315 (4.5)	795 (13.7)	5044 (6.4)
Non–mental health primary diagnosis	8179 (12.4)	1547 (22.3)	437 (7.6)	10 163 (13.0)
Other mental health diagnosis group	7536 (11.5)	2339 (33.8)	810 (14.0)	10 685 (13.6)
Aggressive behavior^c^	16 092 (24.5)	1018 (14.7)	1583 (27.4)	18 693 (23.8)
Prior MBH visit within 1 y^b^	17 895 (27.2)	1037 (15.0)	1461 (25.2)	20 393 (26.0)
Time to first clinician evaluation, median (IQR), min^d^	35.0 (18.0-66.0)	42.0 (19.8-95.0)	40.0 (20.1-79.7)	35.7 (18.0-69.0)
ED length of stay, median (IQR), min	385.2 (240.0-836.0)	269.0 (187.6-390.0)	231.0 (159.4-369.0)	354.0 (225.0-740.0)
Disposition				
Discharged	28 647 (43.6)	5071 (73.2)	4147 (71.7)	37 865 (48.3)
Admission, observation, or died	32 752 (49.9)	1758 (25.4)	1603 (27.7)	36 113 (46.1)
Transferred	4272 (6.5)	95 (1.4)	37 (0.6)	4404 (5.6)
Unknown	24 (0.04)	5 (0.1)	0	29 (0.04)
Return visit within 7 d	2650 (4.0)	327 (4.7)	261 (4.5)	3238 (4.1)
Return visit within 30 d	8649 (13.2)	808 (11.7)	779 (13.5)	10 236 (13.1)
Return MBH visit within 7 d^b^	1846 (2.8)	145 (2.1)	172 (3.0)	2163 (2.8)
Return MBH visit within 30 d^b^	6443 (9.8)	397 (5.7)	543 (9.4)	7383 (9.4)

Abbreviations: ED, emergency department; ESI, Emergency Severity Index; MBH, mental or behavioral health.

^a^ Lower ESI scores indicate higher acuity. *P* < .001 for all comparisons.

^b^ Based on a primary diagnosis in the Child and Adolescent Mental Health Disorders Classification System.

^c^ Based on all diagnoses during the visit.

^d^ When time to first clinician evaluation was greater than 1440 minutes (ie, 24 hours), it was set to 1440 minutes.

### Rates of Overtriage and Undertriage

Heart rate or respiratory rate were missing in 3847 ED visits (4.9%), precluding application of definitions of overtriage and undertriage. Of the remaining 74 564 visits, 25 668 (34.4%) were classified as appropriately triaged, 42 589 (57.1%) as overtriaged, and 6307 (8.5%) as undertriaged (Table 4). Overtriage occurred in 6068 of 10 419 visits (58.2%) by Hispanic patients, 12 992 of 22 359 visits (58.1%) by non-Hispanic Black patients, and 19 828 of 35 221 visits (56.3%) by non-Hispanic White patients and in 40 581 of 70 756 visits (57.4%) by patients with English language preference and 1555 of 2911 visits (53.4%) by patients with Spanish language preference. Undertriage occurred in 849 of 10 419 visits (8.1%) by Hispanic patients, 1851 of 22 359 visits (8.3%) by non-Hispanic Black patients, and 2997 of 35 221 visits (8.5%) by non-Hispanic White patients and in 5902 of 70 756 visits (8.3%) by patients with English language preference and 304 of 2911 visits (10.4%) by patients with Spanish language preference. Rates of appropriate triage among visits by patients from racial and ethnic minority groups and patients who prefer a language other than English varied by site (eFigure 2 in Supplement 1).

**Table 4.** Triage Accuracy of MBH Emergency Department Visits by Visit Characteristics

Characteristic	Triage group, No. (%)^a^			No. overall (N = 74 564)
	Overtriaged (n = 42 589)	Undertriaged (n = 6307)	Appropriate triage (n = 25 668)	
Age, y				
5-9	6541 (81.2)	521 (6.5)	998 (12.4)	8060
10-14	18 856 (53.1)	3069 (8.6)	13 594 (38.3)	35 519
15-17	17 192 (55.5)	2717 (8.8)	11 076 (35.7)	30 985
Sex				
Male	17 822 (61.2)	2510 (8.6)	8797 (30.2)	29 129
Female	24 765 (54.5)	3796 (8.4)	16 869 (37.1)	45 430
Unknown	2 (40.0)	1 (20.0)	2 (40.0)	5
Race and ethnicity				
Hispanic	6068 (58.2)	849 (8.1)	3502 (33.6)	10 419
Non-Hispanic Black	12 992 (58.1)	1851 (8.3)	7516 (33.6)	22 359
Non-Hispanic White	19 828 (56.3)	2997 (8.5)	12 396 (35.2)	35 221
Non-Hispanic other^b^	3183 (55.9)	523 (9.2)	1991 (34.9)	5697
Unknown	518 (59.7)	87 (10.0)	263 (30.3)	868
Preferred language				
English	40 581 (57.4)	5902 (8.3)	24 273 (34.3)	70 756
Spanish	1555 (53.4)	304 (10.4)	1052 (36.1)	2911
Other	375 (49.0)	97 (12.7)	293 (38.3)	765
Unknown	78 (59.1)	4 (3.0)	50 (37.9)	132
Arrival mode				
EMS (ground or air)	9888 (48.9)	1966 (9.7)	8349 (41.3)	20 203
Other	31 674 (60.5)	4166 (8.0)	16 546 (31.6)	52 386
Unknown	1027 (52.0)	175 (8.9)	773 (39.1)	1975
Arrival time				
Daytime (07:00-14:59)	15 763 (60.4)	2202 (8.4)	8154 (31.2)	26 119
Evening (15:00-22:59)	21 886 (56.7)	3103 (8.0)	13 643 (35.3)	38 632
Overnight (23:00-06:59)	4940 (50.3)	1002 (10.2)	3871 (39.4)	9813
Arrival day of week				
Weekday (Monday to Friday)	34 956 (58.1)	4828 (8.0)	20 385 (33.9)	60 169
Weekend (Saturday or Sunday)	7633 (53.0)	1479 (10.3)	5283 (36.7)	14 395
Season of visit				
Non–school month (June to August)	7721 (54.3)	1466 (10.3)	5042 (35.4)	14 229
School month (September to May)	34 868 (57.8)	4841 (8.0)	20 626 (34.2)	60 335
Arrival volume quintile^c^				
Very low	1145 (48.1)	255 (10.7)	979 (41.2)	2379
Low	3209 (52.7)	626 (10.3)	2253 (37.0)	6088
Moderate	7413 (56.8)	1141 (8.7)	4496 (34.5)	13 050
High	12 963 (58.5)	1793 (8.1)	7411 (33.4)	22 167
Very high	17 859 (57.8)	2492 (8.1)	10 529 (34.1)	30 880
Complex chronic condition				
Yes	3446 (46.4)	838 (11.3)	3136 (42.3)	7420
No	39 143 (58.3)	5469 (8.1)	22 532 (33.6)	67 144
Prior MBH visit within 1 y				
Yes	10 785 (56.0)	1456 (7.6)	7022 (36.5)	19 263
No	31 804 (57.5)	4851 (8.8)	18 646 (33.7)	55 301

Abbreviations: EMS, emergency medical services; MBH, mental or behavioral health.

^a^ Percentages are calculated from row totals.

^b^ Includes American Indian or Alaska Native, Asian, Native Hawaiian or Other Pacific Islander, multiracial, and other race or ethnicity.

^c^ Indicates the number of arrivals to the site during the same hour compared with the site-specific hourly median number of visits.

### Visit Characteristics Associated With Overtriage and Undertriage

Associations of sociodemographic and clinical characteristics with overtriage and undertriage after adjusting for visit characteristics, year, and site are presented in the Figure. The adjusted odds of overtriage relative to appropriate triage were higher for visits by patients aged 5 to 9 years (AOR, 4.43; 95% CI, 4.13-4.76) compared with those aged 10 to 14 years; male (AOR, 1.18; 95% CI, 1.14-1.22) compared with female patients; and non-Hispanic Black (AOR, 1.17; 95% CI, 1.12-1.22) compared with non-Hispanic White patients. The adjusted odds of overtriage were lower for visits with a preferred language of Spanish (AOR, 0.90; 95% CI, 0.82-0.99) compared with English, arrival by emergency medical services (AOR, 0.70; 95% CI, 0.67-0.73) compared with other arrival modes, and presence of a complex chronic condition (AOR, 0.70; 95% CI, 0.66-0.74) compared with absence.

**Figure.**
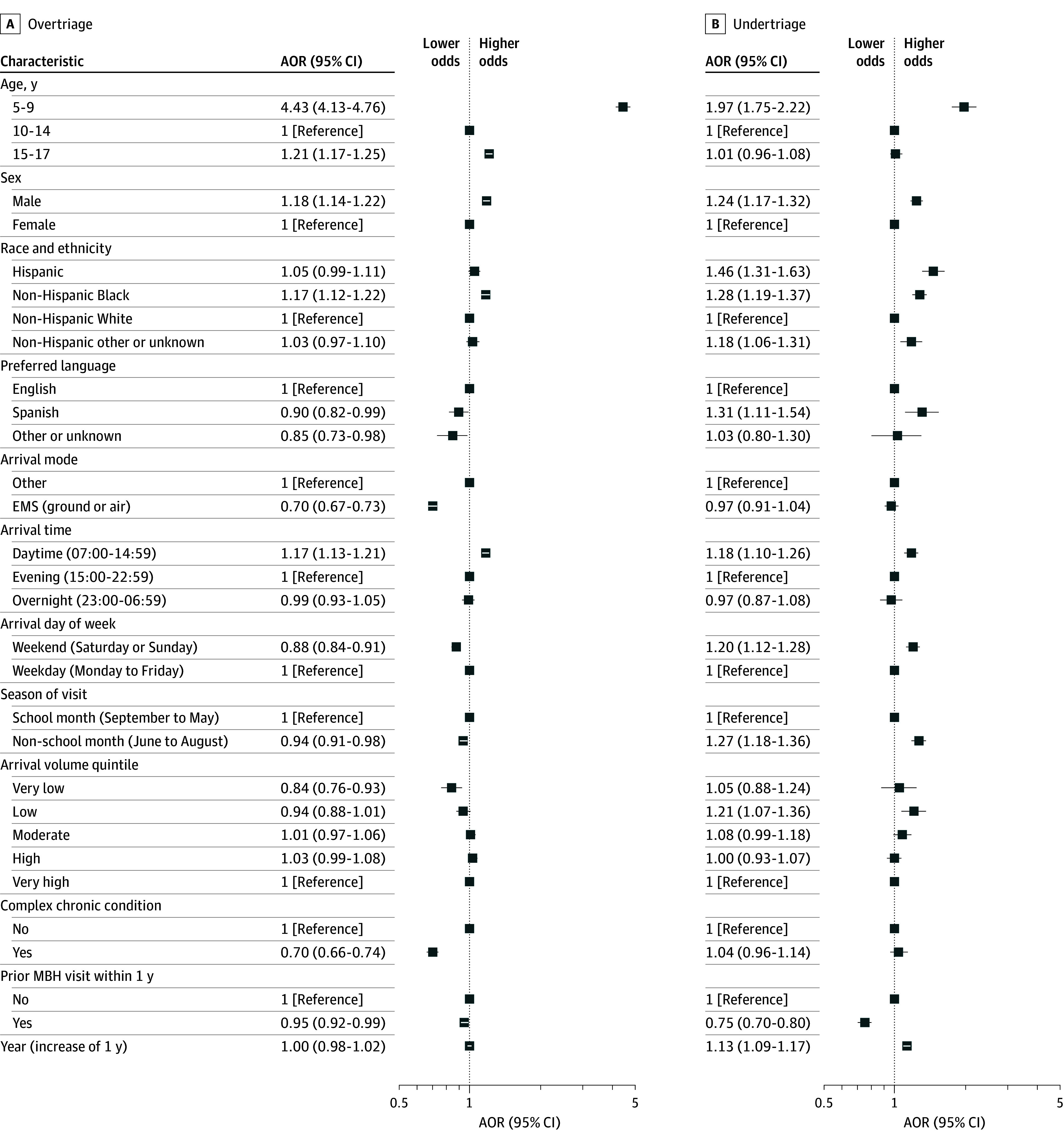
Forest Plot of Characteristics Associated With Overtriage and Undertriage Adjusted odds ratios (AORs) are estimated from multivariable logistic regression models adjusted for visit characteristics, year, and site. Analyses include emergency department visits with known patient sex, heart rate, and respiratory rate. For each variable, the largest category was set as the reference group. EMS indicates emergency medical services; MBH, mental or behavioral health.

The adjusted odds of undertriage relative to appropriate triage were higher for visits by patients aged 5 to 9 years (AOR, 1.97; 95% CI, 1.75-2.22) compared with those aged 10 to 14 years; for visits by male (AOR, 1.24; 95% CI, 1.17-1.32) compared with female patients; for visits by Hispanic (AOR, 1.46; 95% CI, 1.31-1.63) and non-Hispanic Black patients (AOR, 1.28; 95% CI, 1.19, 1.37) and patients of non-Hispanic other or unknown race (AOR, 1.18; 95% CI, 1.06-1.31) compared with non-Hispanic White patients; for visits with Spanish (AOR, 1.31; 95% CI, 1.11-1.54) compared with English language preference; and arrival during a non–school month (AOR, 1.27; 95% CI, 1.18-1.36) compared with a school month. The adjusted odds of undertriage were lower for visits by patients with a prior MBH visit within 1 year (AOR, 0.75; 95% CI, 0.70-0.80).

## Discussion

Within a multicenter pediatric ED data registry, among ED visits by children presenting with MBH symptoms, overtriage occurred in more than half of visits and undertriage occurred in approximately 1 in 12 visits. The likelihood of overtriage and undertriage differed by sociodemographic and clinical characteristics. In adjusted analyses, overtriage was more likely during visits by younger and non-Hispanic Black patients compared with non-Hispanic White patients. Undertriage was more likely among visits by non-Hispanic Black and Hispanic patients compared to non-Hispanic White patients and in visits with a language preference of Spanish relative to English.

Accurate triage systems are needed to match pediatric patients with the right care at the right time, particularly during times of resource strain. Undertriage among children with MBH symptoms may increase the risk of ED safety events, such as elopement, leaving without being seen despite elevated suicide risk, or behavioral dysregulation in waiting areas, potentially resulting in harm to patients, family, or staff. In contrast, overtriage may unnecessarily escalate care for children with MBH symptoms and divert limited resources and attention away from patients with the greatest need. As ED visits by children for MBH symptoms increase over time, straining available resources,^5,21,22^ an ability to accurately distinguish levels of urgency on arrival becomes even more critical.

To our knowledge, this study is the first to examine rates of mistriage among pediatric ED visits for MBH symptoms. Prior studies have identified high rates of mistriage among pediatric ED visits in general. In an analysis of more than 1 million pediatric visits across 21 EDs,^4^ overtriage occurred in 59% and undertriage occurred in 7% of visits. A national sample of US pediatric ED visits with an ESI score of 3 to 5^3^ similarly identified high mistriage rates of 53%, most often due to overtriage, with higher mistriage rates in general EDs than in pediatric EDs. Consistent with our findings, younger age was associated with mistriage of pediatric ED visits,^3^ perhaps because younger children have a reduced ability to communicate presenting symptoms and needs.^4^

Among children presenting to the ED with MBH symptoms, we identified inequities in triage based on race, ethnicity, and language preference, echoing the results of prior research examining pediatric triage in general.^4,9,10,20^ In a large multicenter study and in single-center studies, undertriage was more likely among pediatric ED visits by Black and Hispanic patients compared with White patients.^4,9,20^ Single-center studies have also identified higher rates of undertriage among pediatric ED visits by patients who prefer a language other than English compared with those who prefer English.^9,10^ While our findings were consistent with these studies, we additionally identified a higher likelihood of overtriage of non-Hispanic Black children compared with non-Hispanic White children.

Underlying drivers for inequities in triage may include clinician bias, underutilization of professional interpretation, and systemic racism. Implicit bias, which refers to unconscious stereotypes or attitudes, has been well described among pediatric clinicians, particularly with regard to race and ethnicity.^21,22,23^ During the triage process, explicit or implicit bias might lead clinicians to perceive Black youths as older or more aggressive than they are,^24,25,26^ contributing to overtriage. Additionally, ED clinicians routinely underuse professional interpretation for families who prefer languages other than English,^27,28^ with resulting miscommunication driving mistriage.^29^ Finally, systemic racism, present in society and health care systems, may infiltrate specific health care processes such as triage.^30,31^ Importantly, factors beyond the triage process itself may also contribute to inequities in mistriage rates. Inappropriately high or low use of ED resources among specific patient groups, resulting from biases in care, may also lead to mismatches between assigned triage scores and actual resource use. Future work is needed to elucidate how biases influence patient care throughout the ED care experience from arrival to discharge.

We identified variation in mistriage rates by site, suggesting there may be opportunities for sites to improve accuracy and equity in triage practices. Opportunities may include staff education regarding implicit bias and systemic racism, as well as standardized identification of interpreter needs and increased use of professional interpreters in triage.^32,33^ Additionally, emerging evidence suggests that ED triage tools informed by artificial intelligence may enhance performance and reduce inequities, although careful implementation, prospective evaluation, and explicit attention to equity are needed to avoid reinforcing human biases.^34,35,36^

Our study focused on ESI, the triage system used in more than 90% of US EDs.^2,37^ The ESI Handbook recommends assigning patients to level 2 if patients present a danger to themselves, others, or the environment, but otherwise offers limited guidance on triaging patients with MBH symptoms. In ESI algorithms, MBH-specific resources (eg, one-to-one observation, physical restraint application) are not currently considered in assessments of anticipated ED resource use.^6^ However, other ED triage tools offer more detailed guidance on assessment of patients presenting with MBH symptoms.^38^ For instance, the Australian Mental Health Triage Scale provides detailed descriptions of observed and reported MBH symptoms that map onto 5 categories, enabling more nuanced differentiation of patient need.^39^ In a study that compared ESI with the Australian Triage Scale among 100 adult ED patients with MBH symptoms,^40^ the Australian system was less likely to rate patients as urgent and emergent, with substantially more variation in score assignments than ESI, suggesting an increased ability to distinguish between levels of urgency. In 1 US pediatric ED, when the Australian Triage Scale was implemented alongside staffing changes and environmental modifications,^41^ the ED experienced lower rates of staff injuries, fewer security codes, decreased ED length of stay for MBH visits, and decreased MBH admission rates. Further research is needed to compare the performance of different triage systems for children with MBH symptoms in terms of accuracy, ED operational outcomes, and patient outcomes. Triage systems must be designed, optimized, and implemented to enable accurate and equitable resource allocation for all children.

### Limitations

Because the PECARN Registry is an electronic health record database, there is potential for misclassification of ED visits. Due to large sample sizes, some statistically significant differences may not be clinically significant. Most participating EDs were at children’s hospitals, potentially limiting generalizability of results. There is potential for misclassification of resource types, including consultations and procedures that were identified using NLP, and the resource type of electrocardiogram could not be identified from the dataset. Some visits may have been triaged as high risk due the patient’s risk of danger to themselves, others, or the environment, factors that were not captured in the dataset, potentially contributing to misclassification of some appropriately triaged visits as overtriaged. Information about the demographic characteristics, training, and certification of nurses conducting triage at each site was not available, nor were data about admission type (psychiatric or nonpsychiatric) or transfer location (to a psychiatric or nonpsychiatric facility).

## Conclusions

In this cross-sectional study of ED visits by children presenting to 15 EDs for MBH symptoms, we found that overtriage was common, and rates of mistriage varied by sociodemographic and clinical characteristics, including race, ethnicity, and language. These findings are relevant to preparedness efforts for EDs to manage surges of pediatric MBH presentations. Our results also inform the need to design more accurate, equitable triage systems for pediatric emergency care, with the overall goal of differentiating which children with MBH symptoms need emergent attention from those who can safely wait for care.

## Notes

Supplement 1. **eTable 1.** Definition of High-Risk Vital Signs **eTable 2.** Definition of Emergency Medication **eTable 3.** Definitions of Resource Types **eTable 4.** Frequently Occurring Mental and Behavioral Health Chief Concerns **eTable 5.** Frequency of Use of Resource Types by Emergency Severity Index **eFigure 1.** Inclusion/Exclusion Flow Diagram **eFigure 2.** Triage Accuracy by Race, Ethnicity, and Language as a Function of Site Demographic Composition

Supplement 2.
Nonauthor Collaborators

Supplement 3.
Data Sharing Statement
