# Supplementary material for: Overtriage and Undertriage of Children Presenting to the Emergency Department for Behavioral Health
Source: JAMA Netw Open. 2026 Mar 24;9(3):e263042. doi: 10.1001/jamanetworkopen.2026.3042 (PMC13014168; doi:10.1001/jamanetworkopen.2026.3042)
Supplement: Supplement 1. — eTable 1. Definition of High-Risk Vital Signs eTable 2. Definition of Emergency Medication eTable 3. Definitions of Resource Types eTable 4. Frequently Occurring Mental and Behavioral Health Chief Concerns eTable 5. Frequency of Use of Resource Types by Emergency Severity Index eFigure 1. Inclusion/Exclusion Flow Diagram eFigure 2. Triage Accuracy by Race, Ethnicity, and Language as a Function of Site Demographic Composition [file jamanetwopen-e263042-s001.pdf]

Supplementary Online Content

Hoffmann JA, Foster AA, Rojas CR, et al; PECARN Registry Study Group. Overtriage and undertriage of children presenting to the emergency department for behavioral health. *JAMA Netw Open.* 2026;9(3):e263042. doi:10.1001/jamanetworkopen.2026.3042

eTable 1. Definition of High-Risk Vital Signs

eTable 2. Definition of Emergency Medication

eTable 3. Definitions of Resource Types

eTable 4. Frequently Occurring Mental and Behavioral Health Chief Concerns

eTable 5. Frequency of Use of Resource Types by Emergency Severity Index

eFigure 1. Inclusion/Exclusion Flow Diagram

eFigure 2. Triage Accuracy by Race, Ethnicity, and Language as a Function of Site Demographic Composition

This supplementary material has been provided by the authors to give readers additional information about their work.

© 2026 Hoffmann JA et al. *JAMA Network Open*.

eTable 1. Definition of High-Risk Vital Signs

**Age**	**Heart Rate**	**Respiratory Rate**	**Oxygen Saturation**
< 1 mo	> 190	> 60	< 92% for all ages
1–<12 mo	> 180	> 55	
1–<3 y	> 140	> 40	
3–<5 y	> 120	> 35	
5–12	> 120	> 30	
12–<18 y	> 100	> 20	

© 2026 Hoffmann JA et al. *JAMA Network Open*.

eTable 2. Definition of Emergency Medication

**Emergency Medications**[a]	
**Critical Care**	**Pharmacologic Restraint**
Adenosine	Amobarbital
Aminophylline	Aripiprazole
Amiodarone	Benztropine
Atropine	Chlordiazepoxide
Bicarbonate	Chlorpromazine
Calcium chloride	Dexmedetomidine
Calcium gluconate	Diazepam
Dextrose (D25 or D50)	Diphenhydramine
Dobutamine	Droperidol
Dopamine	Haloperidol
Diazepam	Hydroxyzine
Epinephrine	Ketamine
Fluid boluses, 2 or more weight-based boluses (normal saline, lactated ringers, D5 normal saline, D5 lactated ringers)	Methohexital
Fosphenytoin	Midazolam
Furosemide	Olanzapine
Intravenous insulin	Paliperidone
Magnesium	Pentobarbital
Lorazepam	Phenobarbital
Naloxone	Prochlorperazine
Norepinephrine	Promazine
Phenobarbital	Promethazine
Phenytoin	Risperidone
Prostaglandin E	Thiopental
Solumedrol	Trifluoperazine
Terbutaline	Triflupromazine
Valproate	Ziprasidone

[a] Only medications with a delivery route of intravenous, intramuscular, or intraosseous were included in the definition.

© 2026 Hoffmann JA et al. *JAMA Network Open*.

eTable 3. Definitions of Resource Types

Resource Type	Definition	Count[a]
Medication	At least one medication ordered with intravenous, intramuscular, or inhalation route	1
Intravenous Fluids	At least one order for intravenous fluid	1
Laboratory Test	At least one laboratory test ordered	1
Simple Procedure	At least one simple procedure, defined as incision and drainage, laceration repair, gastrostomy tube replacement, joint aspiration, lumbar puncture, nursemaid's elbow reduction, foreign body removal, or urinary catheterization, as identified from visit narratives using natural language processing	1
Complex Procedure (Sedation)	At least one order for ketamine	2
Radiographs	At least one radiograph ordered	1
Advanced Imaging	At least one radiology test ordered with type of computed tomography, magnetic resonance imaging, ultrasound, or interventional radiology	1
Specialty Consult	At least one specialty consult, as identified from visit narratives using natural language processing. Social work involvement was not considered a specialty consult.	1

[a] Each resource type contributed 1 or 2 points, which were summed to obtain a count of the total number of resource types used during the visit.

© 2026 Hoffmann JA et al. *JAMA Network Open*.

eTable 4. Frequently Occurring Mental and Behavioral Health Chief Concerns

Chief Concern [a]
Psych Evaluation
Ingestion [b]
Behavioral Concern
[Placeholder Indicating Mental or Behavioral Health Visit] [c]
Psychiatric Emergencies
ED Psych
Anxiety
Intentional Ingestion
Suicidal
Alcohol Intoxication
Psychiatric Evaluation
Behavioral Problem
Aggressive Behavior
Suicide Ideation
Eating Disorder
Abnormal Behavior
Suicide Attempt
ED Ingestion [b]
Anorexia
ED Behavioral Health
Suicidal Behavior/Threats
Panic Attack
Poisoning [b]
Disordered Eating
Psychiatric Problem
Assaultive or Suicidal Behavior
Depression
Self Injurious Behavior
Hallucinations
Possible Ingestion [b]

ED: Emergency Department

a. The 30 most frequently occurring mental or behavioral health chief complaints, representing 95% of chief complaints in the study sample, are listed in order of frequency.
b. Chief complaints of ingestion or overdose, without intent specified, were categorized as “mental or behavioral health” if the patient was ≥10 years old.
c. Placeholder was consistently used, only for mental and behavioral health visits, to protect patient privacy.

© 2026 Hoffmann JA et al. *JAMA Network Open*.

eTable 5. Frequency of Use of Resource Types by Emergency Severity Index

**Resource Type**	**Overall** (N = 78411)	**ESI 1 or 2** (N = 65695)	**ESI 3** (N = 6929)	**ESI 4 or 5** (N = 5787)
Laboratory Test	19551 (24.9%)	17543 (26.7%)	1473 (21.3%)	535 (9.2%)
Radiograph	3631 (4.6%)	2943 (4.5%)	469 (6.8%)	219 (3.8%)
Advanced Imaging	1649 (2.1%)	1346 (2.0%)	261 (3.8%)	42 (0.7%)
Intravenous Fluids	6477 (8.3%)	5543 (8.4%)	783 (11.3%)	151 (2.6%)
Intravenous, Intramuscular, or Nebulized Medication	11714 (14.9%)	10219 (15.6%)	1135 (16.4%)	360 (6.2%)
Specialty Consultation	32940 (42.0%)	29222 (44.5%)	2545 (36.7%)	1173 (20.3%)
Simple Procedure	1575 (2.0%)	1377 (2.1%)	126 (1.8%)	72 (1.2%)
Complex Procedure (Procedural Sedation)	95 (0.1%)	84 (0.1%)	7 (0.1%)	4 (0.1%)

ESI: Emergency Severity Index
[a] Advanced imaging was defined as computed tomography, magnetic resonance imaging, ultrasound, or interventional radiology

© 2026 Hoffmann JA et al. *JAMA Network Open*.

eFigure 1. Inclusion-Exclusion Flow Diagram

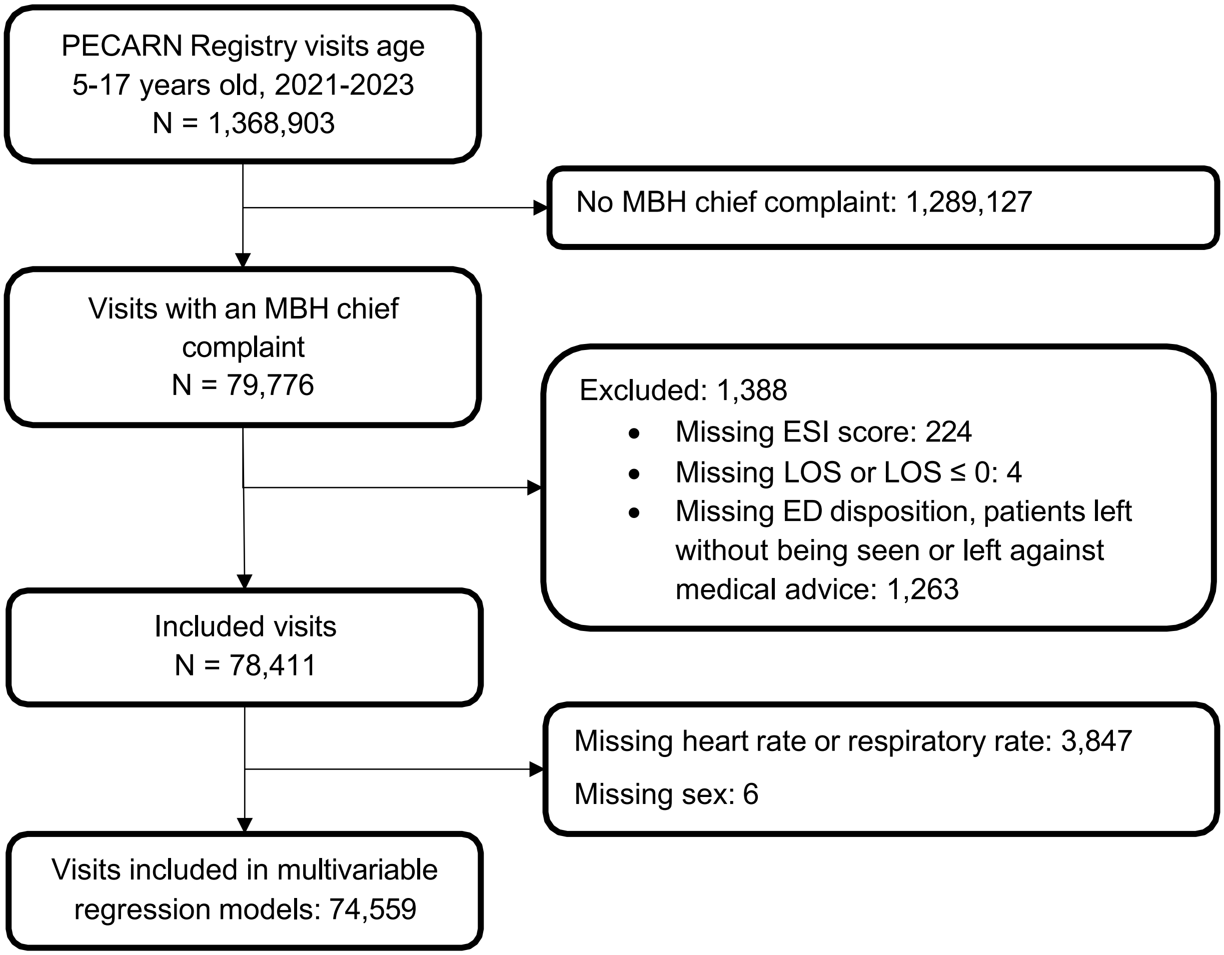

PECARN: Pediatric Emergency Care Applied Research Network; MBH: Mental or behavioral health; ESI: Emergency Severity Index; LOS: Length of Stay; ED: Emergency department

© 2026 Hoffmann JA et al. *JAMA Network Open*.

eFigure 2. Triage Accuracy by Race, Ethnicity, and Language as a Function of Site Demographic Composition

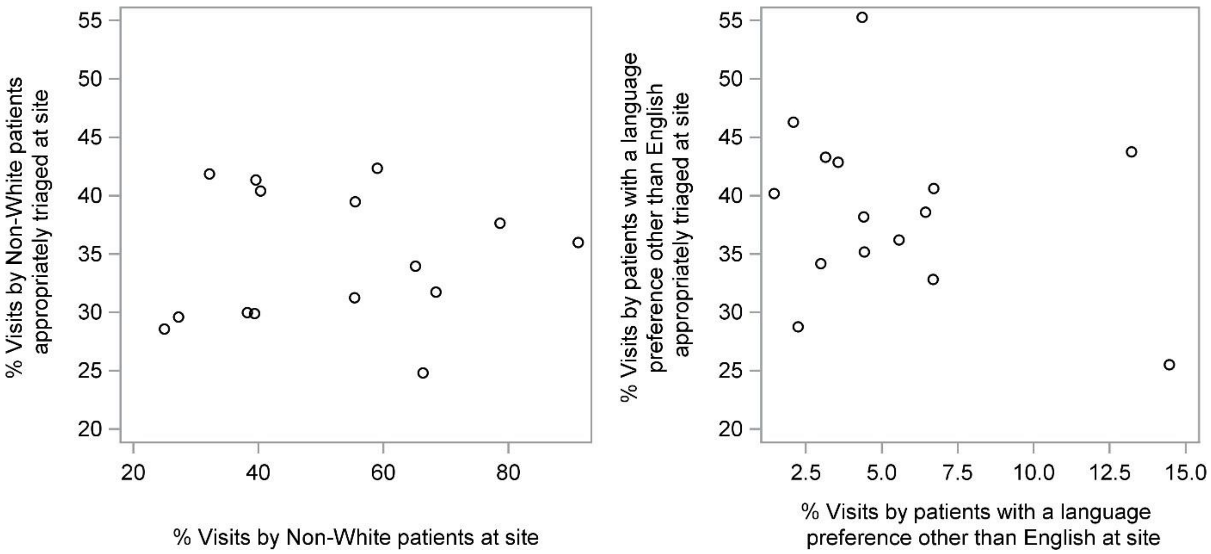

© 2026 Hoffmann JA et al. *JAMA Network Open*.
