# Supplementary material for: Overtriage and Undertriage of Children Presenting to the Emergency Department for Behavioral Health
Source: JAMA Netw Open. 2026 Mar 24;9(3):e263042. doi: 10.1001/jamanetworkopen.2026.3042 (PMC13014168; doi:10.1001/jamanetworkopen.2026.3042)
Supplement: Supplement 2. — Nonauthor Collaborators [file jamanetwopen-e263042-s002.pdf]

Supplemental Online Content: Nonauthor Collaborators
*First name, last name, and suffix (if applicable) are required and will appear in PubMed.

*Group Name(s): PECARN Registry Study Group							
***First Name and Middle Initial(s)**	***Last Name**	***Suffix (eg, Jr, III)**	Academic Degrees	Institution	Location (city, state/province, country)	Role or Contribution, eg, chair, principal investigator	Group (if more than 1 Group listed in the byline) and/or Subgroup (eg, Steering Committee)
Lynn	Babcock		MD, MS	Department of Pediatrics, University of Cincinnati, Cincinnati Children's Hospital Medical Center	Cincinnati, OH	Study Group Member, Site PI	
Lalit	Bajaj		MD, MPH	Department of Pediatrics, Children's Hospital Colorado, University of Colorado School of Medicine	Aurora, CO	Study Group Member, Site PI	
James M.	Chamberlain		MD	Department of Pediatrics, George Washington University, Children's National Hospital	Washington, DC	Study Group Member, Site PI	
Cara	Elsholz		BS	University of Utah School of Medicine	Salt Lake City, UT	Study Group Member, Site PI	
Robert	Grundmeier		MD	Department of Pediatrics, Children's Hospital of Philadelphia, Perelman School of Medicine, University of Pennsylvania	Philadelphia, PA	Study Group Member, Site PI	
Naghma S.	Khan		MD	Department of Pediatrics, Emory University School of Medicine, Children's Healthcare of Atlanta	Atlanta, GA	Study Group Member, Site PI	
Prashant	Mahajan		MD, MPH, MBA	Department of Emergency Medicine, University of Michigan Medical School	Ann Arbor, MI	Study Group Member, Site PI	
Bashar S.	Shihabuddin		MD, MS	Department of Pediatrics, Nationwide Children's Hospital, The Ohio State University College of Medicine	Columbus, OH	Study Group Member, Site PI	

Supplemental Online Content: Nonauthor Collaborators
*First name, last name, and suffix (if applicable) are required and will appear in PubMed.

***First Name and Middle Initial(s)**	***Last Name**	***Suffix (eg, Jr, III)**	Academic Degrees	Institution	Location (city, state/province, country)	Role or Contribution, eg, chair, principal investigator	Group (if more than 1 Group listed in the byline) and/or Subgroup (eg, Steering Committee)
Leah	Tzimenatos		MD	Department of Emergency Medicine, University of California, Davis School of Medicine	Sacramento, CA	Study Group Member, Site PI	
Patrick S.	Walsh		MD, MS	Department of Pediatrics, Medical College of Wisconsin	Milwaukee, WI	Study Group Member, Site PI	
Joseph J.	Zorc		MD, MSCE	Department of Pediatrics, Children's Hospital of Philadelphia, Perelman School of Medicine, University of Pennsylvania	Philadelphia, PA	Study Group Member, Site PI	
