# Supplementary material for: Overtriage and Undertriage of Children Presenting to the Emergency Department for Behavioral Health
Source: JAMA Netw Open. 2026 Mar 24;9(3):e263042. doi: 10.1001/jamanetworkopen.2026.3042 (PMC13014168; doi:10.1001/jamanetworkopen.2026.3042)
Supplement: Supplement 3. — Data Sharing Statement [file jamanetwopen-e263042-s003.pdf]

Data Sharing Statement

Hoffmann. Overtriage and Undertriage of Children Presenting to the Emergency Department for Behavioral Health. *JAMA Netw Open*. Published March 24, 2026. doi:10.1001/jamanetworkopen.2026.3042

Data
Data available: No

Additional Information
Explanation for why data not available: Data will not be shared. A subset of the PECARN Registry is available as a public use dataset at the following website: https://pecarn.org/datasets/.
